# CSF-Progranulin and Neurofilament Light Chain Levels in Patients With Radiologically Isolated Syndrome—Sign of Inflammation

**DOI:** 10.3389/fneur.2018.01075

**Published:** 2018-12-18

**Authors:** Marc Pawlitzki, Catherine M. Sweeney-Reed, Daniel Bittner, Anke Lux, Stefan Vielhaber, Stefanie Schreiber, Friedemann Paul, Jens Neumann

**Affiliations:** ^1^Department of Neurology, Otto-von-Guericke University, Magdeburg, Germany; ^2^Department of Neurology with Institute of Translational Neurology, University Hospital of Muenster, Münster, Germany; ^3^Department for Biometrics and Medical Informatics, Otto-von-Guericke-University, Magdeburg, Germany; ^4^Charité – Universitätsmedizin Berlin, Corporate Member of Freie Universität Berlin, Humboldt-Universität zu Berlin, and Berlin Institute of Health, NeuroCure Clinical Research Center, Berlin, Germany; ^5^Charité – Universitätsmedizin Berlin, Corporate Member of Freie Universität Berlin, Humboldt-Universität zu Berlin, and Berlin Institute of Health, Department of Neurology, Berlin, Germany; ^6^Experimental and Clinical Research Center, Max Delbrueck Center for Molecular Medicine and Charité – Universitätsmedizin Berlin, Corporate Member of Freie Universität Berlin, Humboldt-Universität zu Berlin, and Berlin Institute of Health, Berlin, Germany

**Keywords:** multiple sclerosis, radiologically isolated syndrome, cerebrospinal fluid, neurofilament light chain, progranulin

## Abstract

**Background:** Cerebrospinal fluid (CSF) markers of disease in patients with radiologically isolated syndrome (RIS) are the subject of intense investigation, because they have the potential to enhance our understanding of the natural disease course and provide insights into similarities and differences between RIS and other multiple sclerosis (MS) disease identities.

**Methods:** Here we compared neurofilament light chain (NFL) and progranulin (PGRN) levels in the CSF in RIS patients with levels in patients with different subtypes of MS and healthy controls (HC) using Kruskal–Wallis one-way analysis of variance.

**Results:** Median CSF NFL concentrations in RIS patients did not differ to those in HC and clinically isolated syndrome (CIS) patients, but were significantly lower than in relapsing remitting (RRMS) and primary progressive (PPMS) MS patients. In contrast, RIS patients exhibited higher median CSF PGRN levels than HC and showed no significant differences compared with CIS, RRMS, and PPMS cases.

**Conclusion:** We postulate that elevated PGRN values in the CSF of RIS patients might indicate inflammatory and repair activity prior to axonal disintegration.

## Introduction

Widespread routine clinical implementation of magnetic resonance imaging (MRI) leads to incidental detection of MRI abnormalities suggestive of multiple sclerosis (MS) in patients undergoing cerebral MRI due to non-specific neurological symptoms (e.g., headache, dizziness) (1). Among these patients, a considerable number, mainly young men, develop radiological and clinical progression to relapsing (RRMS) or primary progressive (PPMS) forms of MS within 5 years (2, 3). This observation led to the establishment of the definition of the radiologically isolated syndrome (RIS) (4) as a probable preclinical variant of MS (5, 6). Additionally, a fraction of these patients exhibit oligoclonal bands (OCBs) in the cerebrospinal fluid (CSF), similar to those found in patients with a clinically isolated syndrome (CIS) (7).

Because early disease-modifying treatment (DMT) could delay the conversion from CIS to clinical MS (8), and repeated MRI examinations have led to early identification of progression to MS from CIS, with the offer of more powerful therapies, the question arises as to whether RIS patients could profit from these procedures as well. To address this question, the understanding of pro- and anti-inflammatory activity, repair mechanisms, and axonal loss, in the absence of noticeable clinical events, needs to be expanded to estimate the clinical relevance of incidentally diagnosed MRI lesions.

We consider CSF progranulin (PGRN) and neurofilament light chain (NFL) to serve as *in vivo* measures of inflammatory activity, tissue repair and neuroaxonal damage. In short, in the central nervous system, PGRN is mainly expressed in neurons and microglia (9). Considering anti-inflammatory and repair activity, progranulin (PRGN) has been identified as a molecule, which could regulate inflammation after axonal injury in the context of MS-associated relapses and continuous inflammation in progressive forms of MS by overexpression in activated microglia (10).

Neurofilaments are structural constituents of the neuroaxonal cytoskeleton and integral components of synapses; they are essential for axonal growth, transport, and signaling pathways (11, 12). White matter and cortical injury is related to elevated CSF NFL that represents a downstream effect of neuroaxonal loss (13), and CSF NFL increase has been found in early MS disease stages with axonal injury as well (14).

In the current study, we assessed the concentrations of CSF PGRN and NFL in RIS patients as potential markers of early repair mechanisms/inflammation and axonal loss, to compare them with the CSF PGRN and NFL concentrations in controls and patients at different MS disease stages and with different MS subtypes.

## Methods

### Patients, Controls, and Clinical Assessment

Our cross-sectional study included *n* = 23 RIS patients, diagnosed according to the criteria proposed by Okuda et al. (4) and MAGNIMS (15), and *n* = 15 CIS, *n* = 15 RRMS and n = 26 PPMS patients, diagnosed according to the McDonald criteria (2010) (16). All patients were recruited retrospectively at the Department of Neurology, Otto-von-Guericke University Magdeburg, Germany, between 2012 and 2017. Due to the retrospective character of the study, written informant consent was not obtained, but all analyses were taken from diagnostic procedures in clinical routine.

All patients underwent a lumbar puncture (LP) and their clinical disability was assessed applying the Expanded Disability Status Scale (EDSS) (17). Reasons for performing a MRI examination in RIS patients were non-specific complaints including headache (*n* = 7 [30%]), non-specific dizziness (*n* = 5 [22%]), tinnitus (*n* = 3 [13%]), transitory ischemic attack (5 [22%]), back pain (*n* = 2 [9%]) and idiopathic peripheral facial palsy (*n* = 1 [4%]). In MS patients, disease duration was defined as time in months from symptom onset to the LP, while in RIS cases it was defined as time from the patients' first complaints to the LP. CIS, RRMS and PPMS patients neither presented with a relapse within the last 4 weeks, nor did they received any disease-modifying treatment.

CSF was additionally acquired from a hospital-based cohort of *n* = 30 healthy controls (HC). The CSF from the HC group was obtained from individuals in whom the presence of a neurological disorder had been suspected, but these individuals were deemed to be healthy in retrospect and in particular have normal cerebral MRI scans. In addition to the clinical classification, patients included in the control group also fulfilled the following Reiber laboratory criteria defining a non-inflammatory CSF [<5 cells/μl, >500 mg protein/ml, <2 mmol/l lactate, no disruption of the blood/CSF barrier, no oligoclonal bands (OCB) in the CSF, and no intrathecal immunoglobulin (Ig) G, IgA, or IgM synthesis] (18).

The study was approved by the local ethics committee (No. 07/17).

### Neuro-Imaging Investigations

Brain and spinal cord MRI scans from patients originated from non-standardized protocols from differing MRI units and magnetic field strengths (1.5 or 3.0 Tesla) were performed within 6 months or after CSF measurement. All examinations included T1- and T2-weighted spin-echo sequences with the administration of gadolinium (Gd). Abnormalities including T1-hypointesities, T2-hyperintesities, and Gd-enhanced T1-lesions were initially identified by a neuroradiologist and they were subsequently verified by a MS specialist (M.P.). Brain and spinal cord scans of all RIS cases were reviewed to confirm the fulfillment of dissemination in space (DIS) criteria (4).

### CSF Measures

Immediately after LP, CSF cells were counted, and total protein, albumin quotient (Q_alb_), and OCBs were measured. The remaining CSF material was centrifuged at 4°C, aliquoted, and stored at −80°C until PGRN and NFL analysis was performed. PGRN and NFL levels were measured using commercially available ELISA kits (PGRN: Human Progranulin ELISA kit, Mediagnost, Reutlingen, Germany; NFL: UmanDiagnostics NF-light®, Umeå, Sweden) following the instructions provided by the manufacturer. All samples were processed in duplicate, in serial procedures, and the mean was taken for statistical analysis.

### Statistical Analysis

Statistical analysis was conducted using SPSS 21 (IBM, Armonk, New York, USA). Comparisons of categorical variables (e.g., sex or OCBs) were performed using a α2-test. Moreover, a univariate analysis of variance, including the estimated marginal means was performed to evaluate between-subject-effects and the effect of age, sex on PGRN and NFL. For further group comparisons of continuous variables (e.g., age, CSF NFL, CSF PGRN, disease duration, EDSS), a Kruskal–Wallis one-way analysis of variance was conducted with group (HC vs. RIS vs. CIS vs. RRMS vs. PPMS patients) as the independent variable, applying pairwise *post-hoc* testing (Dunn-Bonferroni-test).

## Results

### Cohort Characterization and MRI Examination

The demographics, the clinical and MRI data of the cohorts are provided in Table 1. Median age and sex [χ^2^(4) = 0.07] did not differ between SC, RIS, CIS, and RRMS patients, whereas PPMS patients were significantly older than SC, RIS (*p* < 0.001, respectively) and RRMS patients (*p* = 0.002). Median disease duration was longer in PPMS compared to RIS and CIS (*p* < 0.001, respectively). Median EDSS at the time of LP differed between RIS compared to RRMS and PPMS (*p* = 0.007; *p* < 0.001), as well as between CIS and PPMS (*p* < 0.001) and RRMS vs. PPMS (*p* = 0.02).

**Table 1:** Summary of the clinical and radiologic data of all investigated groups.

	**HC (*N* = 30)**	**RIS (*N* = 23)**	**CIS (*N* = 15)**	**RRMS (*N* = 15)**	**PPMS (*N* = 26)**
Age at lumbar puncture (years)	35 (18-49)	37 (18–72)	44 (21–61)	33 (17–55)	52 (25-71)
Male SEX, *N* (%)	12 (33)	8 (35)	3 (16)	6 (30)	15 (60)
Disease duration (months)	–	6 (0–89)	4 (1–78)	19 (2–174)	61 (12–255)
EDSS	–	0 (0–1.5)	1 (0–3.5)	1.5 (0–6)	4.5 (2–7.5)
Presence of Gd+ lesions, *n* (%)	–	0 (0)	0 (0)	4 (29)	3 (11.5)
Presence of periventricular lesions, *n* (%)	–	22 (96)	13 (87)	13 (87)	25 (96)
Presence of infratentorial lesions, *n* (%)	–	9 (39)	3 (20)	7 (46)	16 (62)
Presence of juxtacortical lesions, *n* (%)	–	14 (61)	8 (53)	10 (67)	20 (77)
Presence of spinal cord lesions lesions, *n* (%)	–	4 (17)	5 (33)	8 (53)	20 (77)

*N, number of participants; unless otherwise reported median (range) is given. CSF, cerebrospinal fluid; CIS, Clinically isolated syndrome; EDSS, Expanded Disability Status Scale; Gd+, Gadolinium enhancing; HC, healthy controls; PPMS, primary progressive multiple sclerosis; RIS, radiologically isolated syndrome; RRMS, relapsing remitting multiple sclerosis. Disease duration was defined as the timespan between symptom onset and the date of lumbar puncture*.

### CSF Examination

CSF cell count was significantly higher in RRMS compared with PPMS patients and HC as well as between RIS and HC patients. OCBs were present exclusively in the CSF of 19 (83%) RIS, 25 (96%) PPMS, and all (100%) CIS and RRMS patients (Table 2). Univariate analysis underlined the group difference in particular, and the absence of an effect of age and sex on PGRN and NFL levels.

**Table 2:** Summary of the cerebrospinal fluid results of all investigated groups.

**HC (*N*** **= 30)**	**RIS (*N*** **= 23)**	**CIS** **(*N* = 15)**	**RRMS** **(*N* = 15)**	**PPMS** **(*N* = 26)**	***p*****-values**									
						**HC vs. RIS**	**HC vs. CIS**	**HC vs. RRMS**	**HC vs. PPMS**	**RIS vs.** **CIS**	**RIS vs. RRMS**	**RIS vs.** **PPMS**	**CIS vs. RRMS**	**CIS vs. PPMS**	**RRMS vs. PPMS**
CSF Cell count /μl	1 (0–4)	3 (0–19)	3(1-25)	4(1-16)	1 (0–14)	**0.001**	0.08	**0.01**	1.0	1.0	1.0	0.09	1.0	1.0	**0.04**
CSF protein (mg/dl)	311 (178–460)	371 (238–909)	321 (226–491)	421 (255–650)	410 (248–1047)	0.2	1.0	**0.04**	**0.001**	1.0	1.0	1.0	1.0	1.0	1.0
Positive OCB, N (%)	0 (0)	19 (83)	15 (100)	15 (100)	25 (96)	–	–	–	–	–	–	–	–	–	–
Qalb	4.3 (2.3–6.4)	4.7 (2.8–11.8)	4.03 (3.0–7.2)	5.5 (2.5–8.9)	5.0 (2.0–18.8)	0.05	0.05	0.05	0.05	0.05	0.05	0.05	0.05	0.05	0.05
CSF NFL (pg/ml)	742 (357–1424)	914 (306–2552)	1206 (628–2762)	1893 (364–6458)	1699 (990–4275)	1.0	**0.03**	**<0.001**	**<0.001**	0.3	**<0.001**	**<0.001**	0.8	0.5	1.0
CSF PGRN (pg/ml)	0.67 (0.43–1.12)	0.82 (0.50–1.20)	0.80 (0.60–1.45)	0.80 (0.63–1.30)	0.91 (0.52–1.59)	**0.004**	**0.04**	**0.004**	** < 0.001**	1.0	1.0	0.7	1.0	0.6	1.0

*N, number of participants; unless otherwise reported median (range) is given. CIS, clinically isolated syndrome; CSF, cerebrospinal fluid; HC, healthy controls; NFL, neurofilament light chain; PGRN, progranulin; PPMS, primary progressive multiple sclerosis; Qalb, albumin quotient; RIS, radiologically isolated syndrome; RRMS, relapsing remitting multiple sclerosis. Disease duration was defined as the timespan between symptom onset and time of lumbar puncture. For group comparisons a Kruskal–Wallis one-way analysis of variance with post-hoc Dunn-Bonferroni-testing were conducted. Bold value indicates P-values < 0.05 were deemed to be statistically significant*.

**Figure 1:**
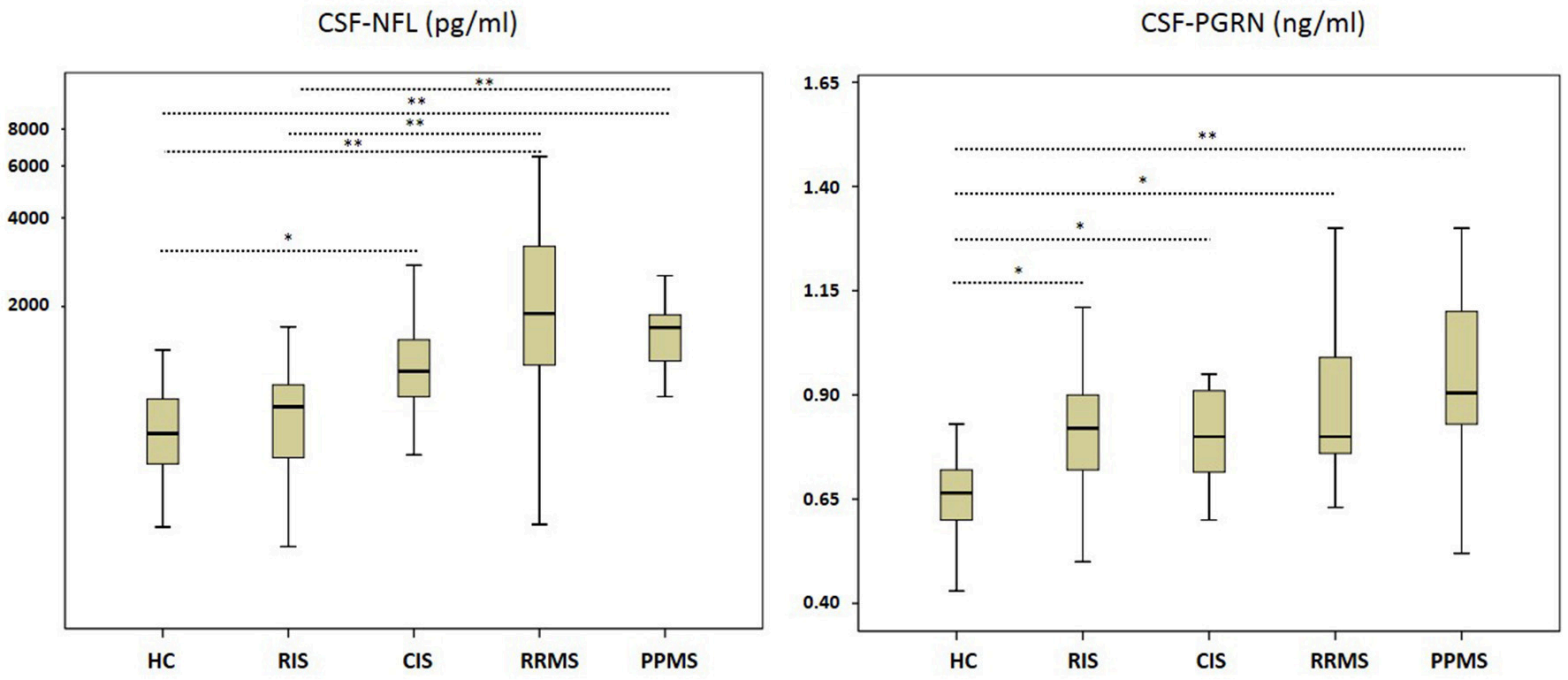
Neurofilament light (NFL) and Progranulin (PGRN) in cerebrospinal fluid (CSF). CIS, clinically isolated syndrome; HC, healthy controls; PPMS, primary progressive multiple sclerosis; RIS, radiologically isolated syndrome; RRMS, relapsing remitting multiple sclerosis. Boxes indicate the interquartile range, bars indicates median CSF-NFL/PGRN values, and Whiskers present the 95% Cl. Group comparisons were conducted using a Kruskal–Wallis one way analysis of variance with *post-hoc* Dunn–Bonferroni-testing. *P* < 0.05 were deemed to be statistically significant. RIS, CIS, RRMS, and PPMS showed higher CSF PGRN values than HC, while PPMS and RRMS also differed in contrast to RIS cases. RJS, CIS, RRMS, and PPMS showed higher CSF PGRN values than HC. ^*^P < 0.005; ^**^P < 0.001.

The concentration of CSF NFL was significantly higher in CIS, RRMS, and PPMS compared to RIS and HC, while there were no significant differences between RIS and HC (Figure 1 and Table 2). The comparison of CSF PRGN between groups revealed significantly higher levels in CIS, RIS, RRMS, and PPMS than detected in HC (Figure 1 and Table 2).

## Discussion

Detection and characterization of early biomarkers in RIS patients, in order to investigate whether they could reflect MS disease courses, is an ongoing challenge. We compared the concentrations of CSF PGRN and NFL in RIS patients with the concentrations in healthy controls and in patients at different disease stages and with different subtypes of treatment-naive MS in the absence of an acute clinical relapse. Our analysis revealed similar PGRN concentrations in RIS patients to those found in several MS subtypes. PGRN levels were significantly higher, on the other hand, in RIS patients than in HC, while NFL concentrations did not differ between the RIS and HC cohorts.

To the best of our knowledge, the current study provides the largest comparison of CSF PGRN levels in different subtypes of MS, including RIS patients, to date. Surprisingly, while NFL values in the CSF of RIS patients were comparable with the HC cohort and significant lower compared with those in patients at different MS disease stages, the RIS cohort exhibited significantly higher concentrations of PRGN in comparison with the HC cohort and showed similar concentrations to those found in patients with the different MS subtypes.

Former studies have reported elevated (19) or unchanged CSF PGRN values in MS but included patients who had experienced an acute relapse (20). The absence of differences of CSF PGRN thus seems to be unusual, because acute inflammation is considered to provoke PGRN expression (19). Furthermore, it has been shown that disease-modifying treatment could decrease PGRN levels (19). In order to exclude heterogeneity, we divided the cohorts into distinct subtypes (RIS, CIS, RRMS, PPMS), comprising only patients without an acute clinical relapse and also without disease-modifying treatments.

The role of PGRN is currently under intense investigation, and microglia cells have been identified as expressing and secreting PGRN after axonal injury (21). CSF PGRN level seems to be largely unaffected by blood PGRN concentration and in turn potential blood-CSF barrier disruption which underline the specificity of intrathecal produced PRGN (22). In addition, PGRN levels correlate with the concentration of the proinflammatory cytokine interleukin 6, which is elevated after acute (19) and chronic axonal injury (10). However, NFL levels were not increased in our RIS cohort, indicating that PGRN could be secreted even before axonal injury is detectable in the CSF via elevated NFL concentrations. This finding leads us to speculate tentatively that PGRN may be upregulated early, at the beginning of disease activity, and that RIS might in turn be a prodromal stage of MS. PGRN may thus shed new light on, clinically silent, disease-related alterations at the earliest MS stages. In line with histological findings that inflammation is the primary hallmark of MS and induces neuronal injury (23, 24), we suggest that PGRN might be more sensitive to detecting early disease-related abnormalities, also in RIS cases, in the face of (still) normal axonal integration, as measured by NFL levels (25).

The finding of missing NFL elevation in RIS patients is surprising, given the early, MRI-detectable neurodegeneration already found in the RIS cohorts (26) and the recognized association between raised CSF NFL and GD-enhancing white matter lesions in RIS patients (27). However, no GD-enhancing white matter lesions were present in our RIS group, suggesting that our cohort is more homogeneous or less severely affected, and acute axonal loss might thus play an insignificant role and be therefore not detectable via the elevated NFL concentrations that are seen in relapsing and progressive MS patients (14, 28, 29).

In addition to the MRI findings resulting in a diagnosis of RIS, we identified CSF abnormalities, e.g., OCBs in the CSF, in almost all of our RIS patients, which is in line with previous studies, in which OCBs were identified as independent predictors for early conversion to MS (27, 30). Our RIS-cohort may thus be characterized as high-risk patients (27) for conversion. However, the prevalence of OCBs is not specific for MS (31), nor does it mirror acute or continuous inflammatory activity (32).

Here we demonstrated significantly elevated CSF PGRN levels in RIS and MS patients during the clinically silent or non-relapsing phase, presumably suggesting ongoing inflammation, while only the later disease stages revealed an increased CSF NFL, thus mirroring axonal injury in addition. We here report the results from a pilot study. The limitations are the relatively small sample size and the higher mean age of PPMS patients. Standardized MRI, in addition, might assist to improve the evaluation of the relationship between white matter lesion volume and CSF-PGRN in MS patients. Hence, longitudinal studies with a larger sample size are needed to overcome these limitations and to determine the prognostic role of PGRN in MS and in particular in RIS patients.

## Ethics Statement

The study was approved by the local ethics committee, Otto-von-Guericke-University, Magdeburg, Germany (No. 07/17).

## Author Contributions

All authors listed have made a substantial, direct and intellectual contribution to the work, and approved it for publication.

### Conflict of Interest Statement

MP received speaker honoraria from Roche, Genzyme, and Novartis as well as travel, accommodation, and meeting expenses from Novartis, Biogen Idec, Genzyme, and MERCK Serono. DB has received honoraria from Bristol-Myers Squibb. JP has received honoraria and research support from Alexion, Bayer, Biogen, Chugai, MerckSerono, Novartis, Genyzme, MedImmune, Shire, Teva, and serves on scientific advisory boards for Alexion, MedImmune, and Novartis. He has received funding from Deutsche Forschungsgemeinschaft (DFG Exc 257), Bundesministerium für Bildung und Forschung (Competence Network Multiple Sclerosis), Guthy Jackson Charitable Foundation, EU Framework Program 7, National Multiple Sclerosis Society of the USA. The remaining authors declare that the research was conducted in the absence of any commercial or financial relationships that could be construed as a potential conflict of interest.
